# Lack of K-Dependent Oxidative Stress in Cotton Roots Following Coronatine-Induced ROS Accumulation

**DOI:** 10.1371/journal.pone.0126476

**Published:** 2015-05-08

**Authors:** Zhiyong Zhang, Xin Zhang, Zebing Hu, Sufang Wang, Jinbao Zhang, Xiaojing Wang, Qinglian Wang, Baohong Zhang

**Affiliations:** 1 Henan Collaborative Innovation Center of Modern Biological Breeding, Henan Institute of Science and Technology, Xinxiang, 453003, China; 2 Department of Biology, East Carolina University, Greenville, NC, 27858, United States of America; University of Arkansas for Medical Sciences; College of Pharmacy, UNITED STATES

## Abstract

Coronatine [COR] is a novel type of plant growth regulator with similarities in structure and property to jasmonate. The objective of this study was to examine the relationship between increased root vitality induced by 10nM COR and reactive oxygen species scavenging under potassium (K)-replete (2.5mM) and K-deficient (0.05mM) conditions in hydroponic cultured cotton seedlings. K-replete and K-deficient conditions increased root vitality by 2.7- and 3.5-fold, respectively. COR treatment significantly decreased lipid peroxidation in cotton seedlings determined by reduction in MDA levels. These results suggest that COR improves the functioning of both enzymatic and non-enzymatic antioxidant systems. Under K-replete and K-deficient conditions, COR significantly increased the activities of antioxidant enzymes SOD (only for K-repletion), CAT, GPX, and APX comparing; COR also significantly increased DPPH-radical scavenging activity. However, COR led to 1.6- and 1.7-fold increases in superoxide anion (O_2_^•-^) concentrations, and 5.7- and 2.1-fold increases in hydrogen peroxide (H_2_O_2_) levels, respectively. Additionally, COR intensified the DAB staining of H_2_O_2_ and the NBT staining of O_2_^•-^. Therefore, our results reveal that COR-induced ROS accumulation stimulates the activities of most antioxidant enzymes but does not induce oxidative stress in cotton roots.

## Introduction

Environmental biotic and abiotic stresses affect plant metabolism, growth and productivity. During stress, electrons at high energy-states are transferred to molecular oxygen (O_2_), resulting in reactive oxygen species (ROSs), including hydrogen peroxide (H_2_O_2_) and superoxide (O_2_^•-^) and hydroxyl (•OH) radicals, which are toxic to plants at high concentrations [1,2]. Among the peroxides, H_2_O_2_ is the most widely spread molecule involving in a broad range of physiological processes, such as growth, development and senescence [3]. For protecting the oxidative stress induced by biotic and abiotic factors, plants have evolved many resistance mechanisms to prevent ROS toxicity, including antioxidant systems that are comprised of different non-enzymatic antioxidant compounds, including carotenoids, glutathione, ascorbic acid and vitamin E, and antioxidant enzymes, for example superoxide dismutase (SOD; EC1.15.1.1), catalase (CAT; EC1.11.1.6), guaiacol peroxidase (GPX; EC1.11.1.7), ascorbic acid peroxidase (APX; EC1.11.1.11) and glutathione reductase (GR; EC1.6.4.2). SOD is responsible for the detoxification of O_2_^•-^ into H_2_O_2_ and O_2_ [4], and CAT and a variety of peroxidases, including GPX and APX, collaborate with GR in the Halliwell–Asada cycle to metabolise H_2_O_2_ [5,6]. Potassium (K) is an important macronutrient affecting plant growth and development, yield, and resistance to environmental stresses [7,8]. K deficiency has been shown to increase H_2_O_2_ production and up-regulate the peroxidase gene expression in *Arabidopsis* thaliana and tomato roots [9–11]. Therefore, antioxidant enzymes were up-regulated under oxidative stress in response to potassium deficiency.

Coronatine [12], which is a novel type of plant growth regulator that possesses similarities in structure and properties to jasmonate, has been shown to significantly stimulate the production of secondary metabolites compared with jasmonate [13]. Several studies have suggested the potential application of COR in the regulation of stress resistance in crops, e.g., increasing drought resistance in rice and upland rice [14] and winter wheat [15] and improving salt tolerance in cotton [16].

Cotton is an important economic crop worldwide, and these plants undergo premature senescence in response to K deficiency [17]. Previous experiments have shown that COR enhances lateral root formation, K uptake and seedling growth in cotton plants [18] and root vitality regardless of the presence of K-sufficient or K-deficient conditions. However, the positive role of COR in root vitality under non-stress or under K-deficient conditions in ROS and antioxidant non-enzymatic and enzymatic systems remains unclear. The objective of this work was to examine the effect of COR on the biochemical defence mechanism of cotton under K deficiency stress.

## Results

### Effects of COR on root vitality under high K and low K

**Fig 1:**
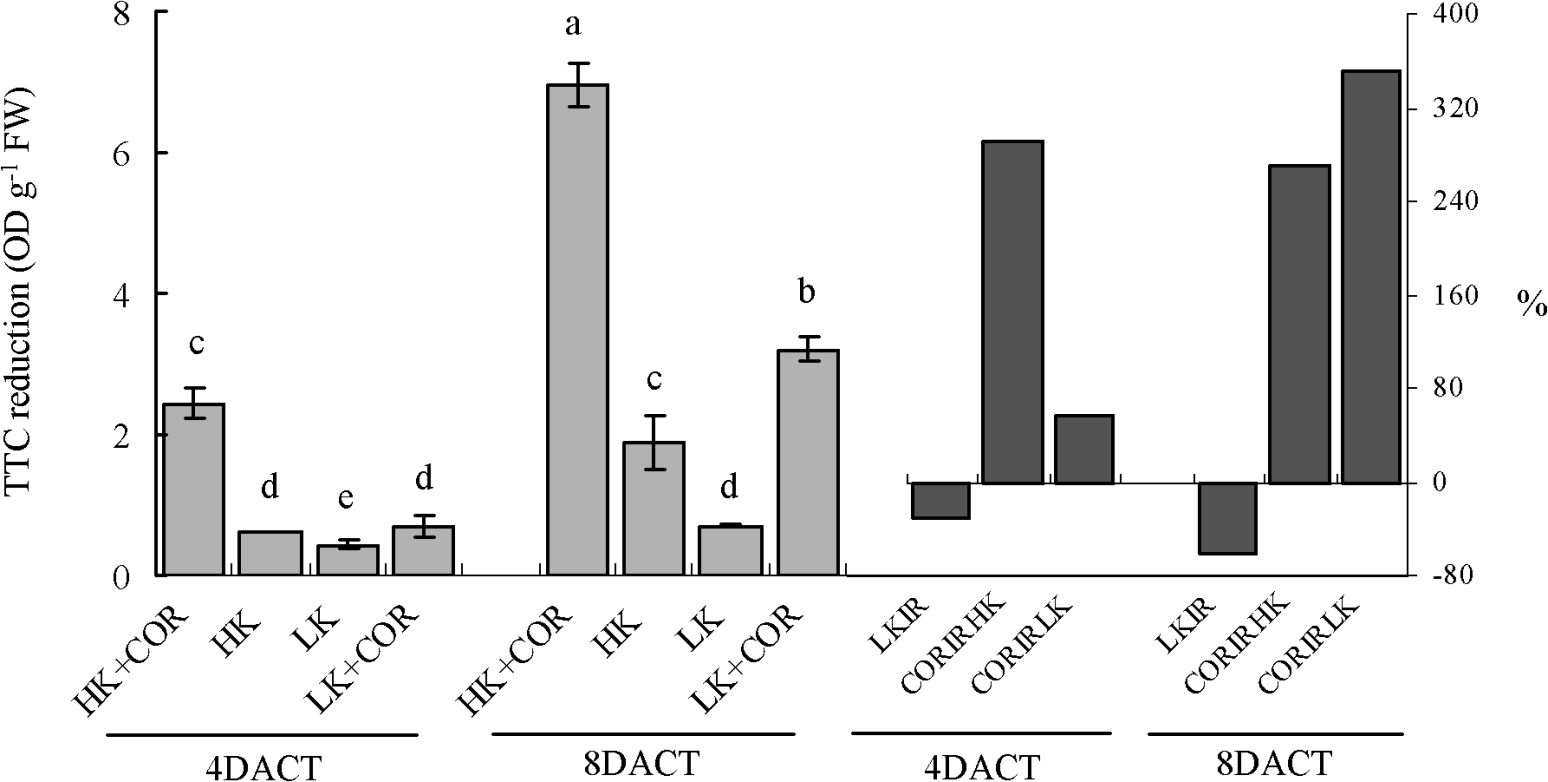
Effects of COR treatment on TTC reduction under high K (2.5 mM) or under low K (0.05 mM) conditions. Emerging seedlings were grown in culture solutions for 3DAKT and subsequently transferred to fresh solutions without (0 nM) or with (10 nM) COR. Levels of TTC reduction were determined at 4DACT (7DAKT) and 8DACT (11DAKT). Values showed by the left subfigure are presented as mean ± SD, n = 5. Bars with same letter are not significantly different at p≤0.05 as determined using Duncan’s multiple range test. Values showed by the right subfigure were, respectively, LK induced rangeability (LKIR) in comparison with HK, COR induced rangeability under HK(CORIRHK) and COR induced rangeability under LK (CORIRLK).COR: coronatine; DAKT: days after K treatment; DACT: days after coronatine treatment; HK: high K; LK: low K.

Root vitality was illustrated as the level of TTC reduction in the root. Under only HK (high K), TTC reduction level was significantly and obviously lower at 7DAKT than that at 11DAKT. Under LK (low K), TTC reduction level was also significantly lower at 7DAKT but were only slightly less than that at 11DAKT. Prolonged K treatment from 7 to 11DAKT led to a significant reduction in TTC reduction level from 31% to 62% under LK compared with HK. From 4DACT to 8DACT, COR treatment significantly increased the TTC reduction level by 2.9- and 2.7-fold under HK and by 0.6- and 3.5-fold under LK, respectively, compared with non-COR treatment, indicating low response rate to COR under LK, compared with HK (Fig 1).

#### Effects of COR on lipid peroxidation and cell membrane damage under HK and LK

**Fig 2:**
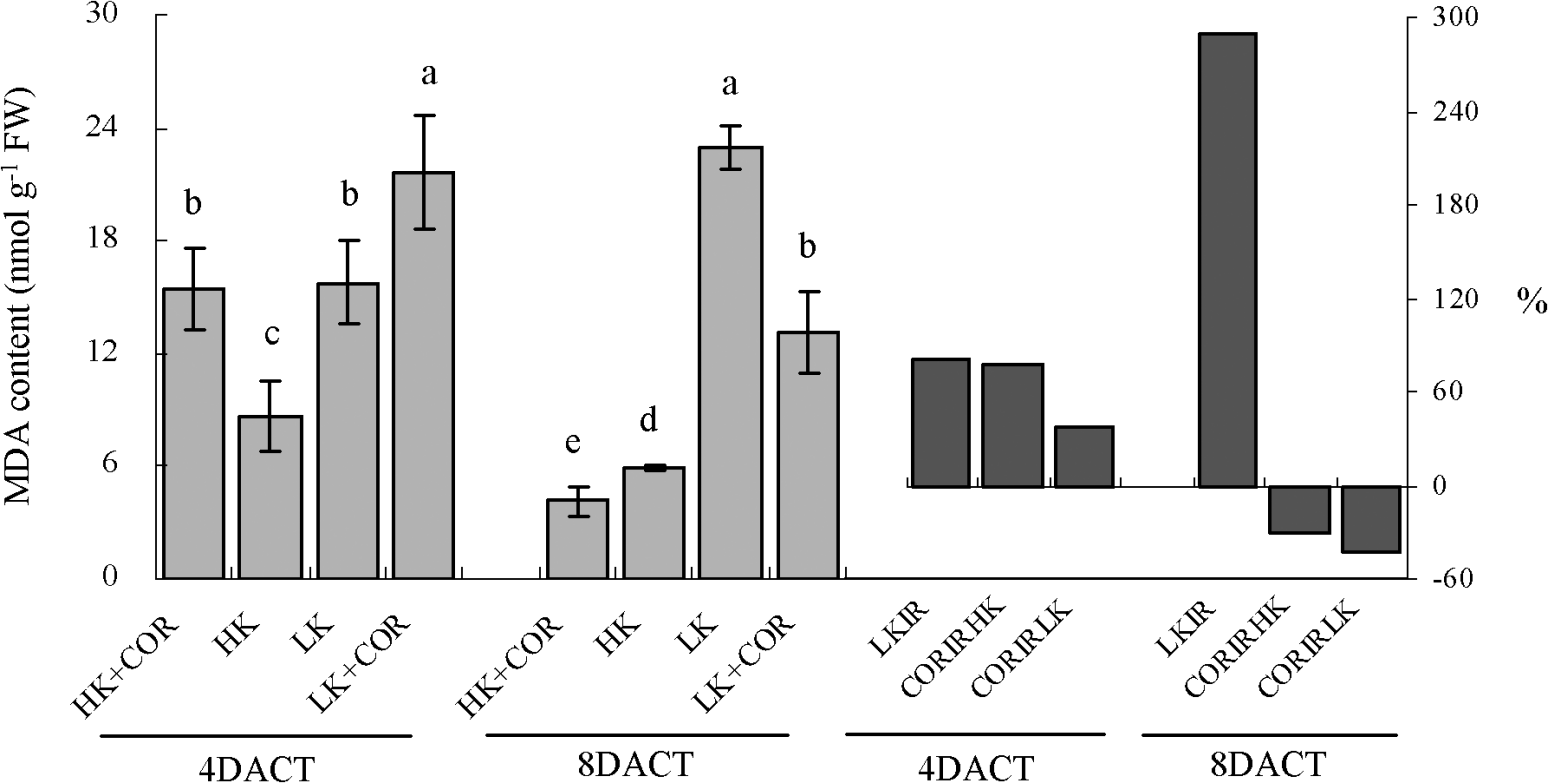
Effects of COR treatment on MDA levels in roots under high K (2.5 mM) or low K (0.05 mM) conditions. Emerging seedlings were grown in solution for 3DAKT and subsequently transferred to fresh solutions without (0 nM) or with (10 nM) COR. MDA levels in roots were determined at 4DACT (7DAKT) and 8DACT (11DAKT). Values are presented as means ± SD, n = 5. Bars with same letter are not significantly different at p≤0.05 as determined using Duncan’s multiple range test. Values showed by the right subfigure were, respectively, LK induced rangeability (LKIR) in comparison with HK, COR induced rangeability under HK(CORIRHK) and COR induced rangeability under LK (CORIRLK).

The MDA levels in the roots were measured to determine the extent of lipid peroxidation. Under HK, MDA concentrations were significantly higher at 7DAKT than at 11DAKT, and the reverse effect was observed under LK. Prolonged K treatment from 7 to 11DAKT led to increased levels of MDA from 82% to 289% under LK compared with HK. COR treatment significantly increased MDA levels by 77% under HK and by 37% under LK at 4DACT. However, COR treatment significantly decreased MDA levels by 30% under HK and by 43% under LK (Fig 2).

**Fig 3:**
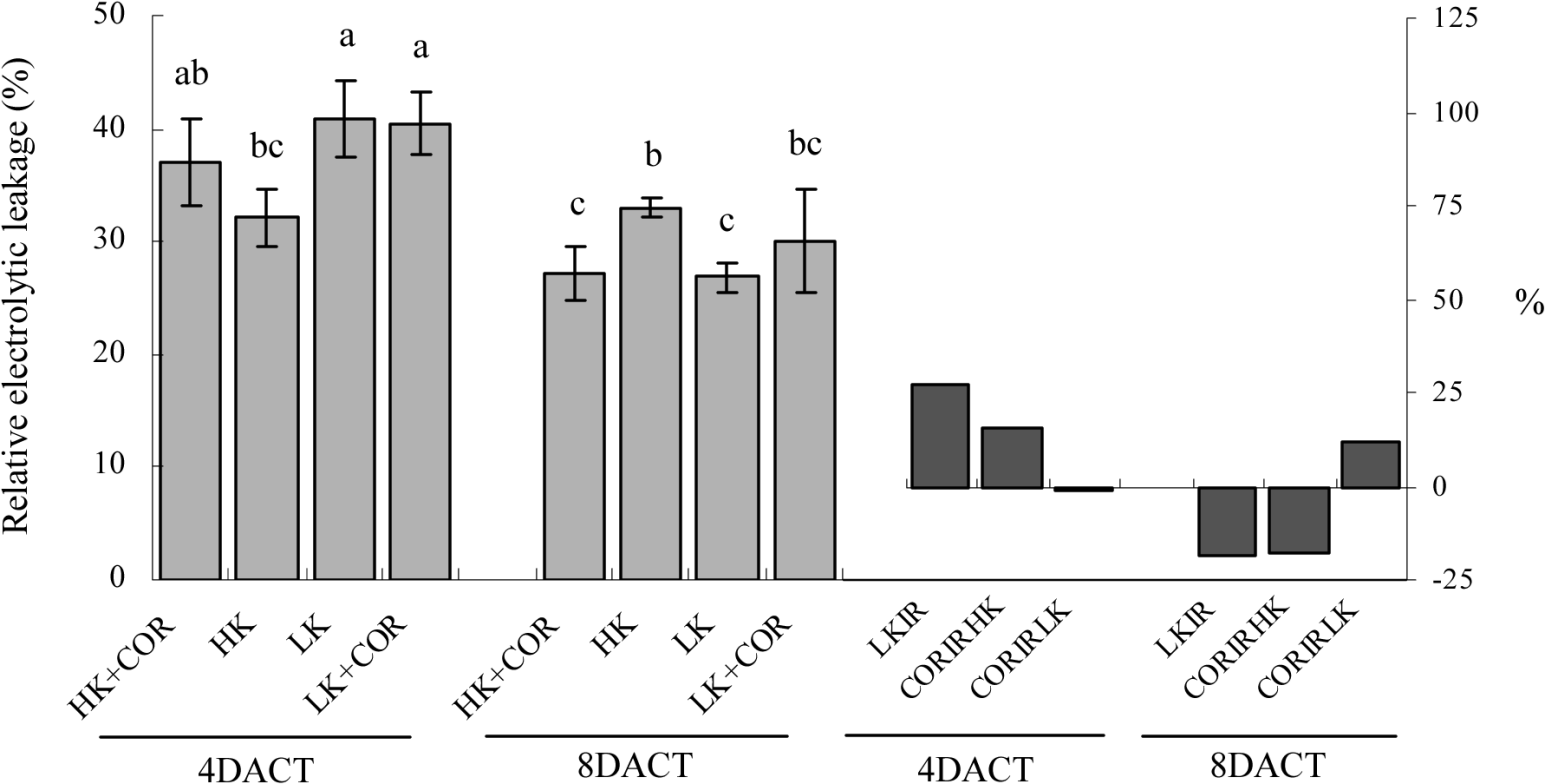
Effects of COR treatment on electrolyte leakage in roots under high K (2.5 mM) or low K (0.05 mM) conditions. Emerging seedlings were grown in solution for 3DAKT and subsequently transferred to fresh solutions without (0 nM) or with (10 nM) COR. Degree of electrolyte leakage in roots was determined at 4DACT (7DAKT) and 8DACT (11DAKT). Values are presented as means ± SD, n = 5. Bars with same letter are not significantly different at p≤0.05 as determined using Duncan’s multiple range test. Values showed by the right subfigure were, respectively, LK induced rangeability (LKIR) in comparison with HK, COR induced rangeability under HK(CORIRHK) and COR induced rangeability under LK (CORIRLK).

The relative electrolytic leakage was determined to assess the degree of cell membrane damage. Compared with HK, LK significantly increased electrolyte leakage by 27% at 7DAKT and significantly decreased leakage by 19% at 11DAKT. COR treatment did not significantly affect electrolyte leakage under either HK or LK with the exception of a significant decrease that was observed at 8DACT under HK (Fig 3). These results indicated that short-term K deficiency enhanced the degree of cell membrane damage, and long-term K deficiency instead reduced it, and that COR lowered the degree of cell membrane damage only at the conditions of HK and relatively long-term treatment.

### Effects of COR treatment on ROS production under HK and LK

#### The levels of O_2_^•-^ and H_2_O_2_ were determined in this experiment

**Fig 4:**
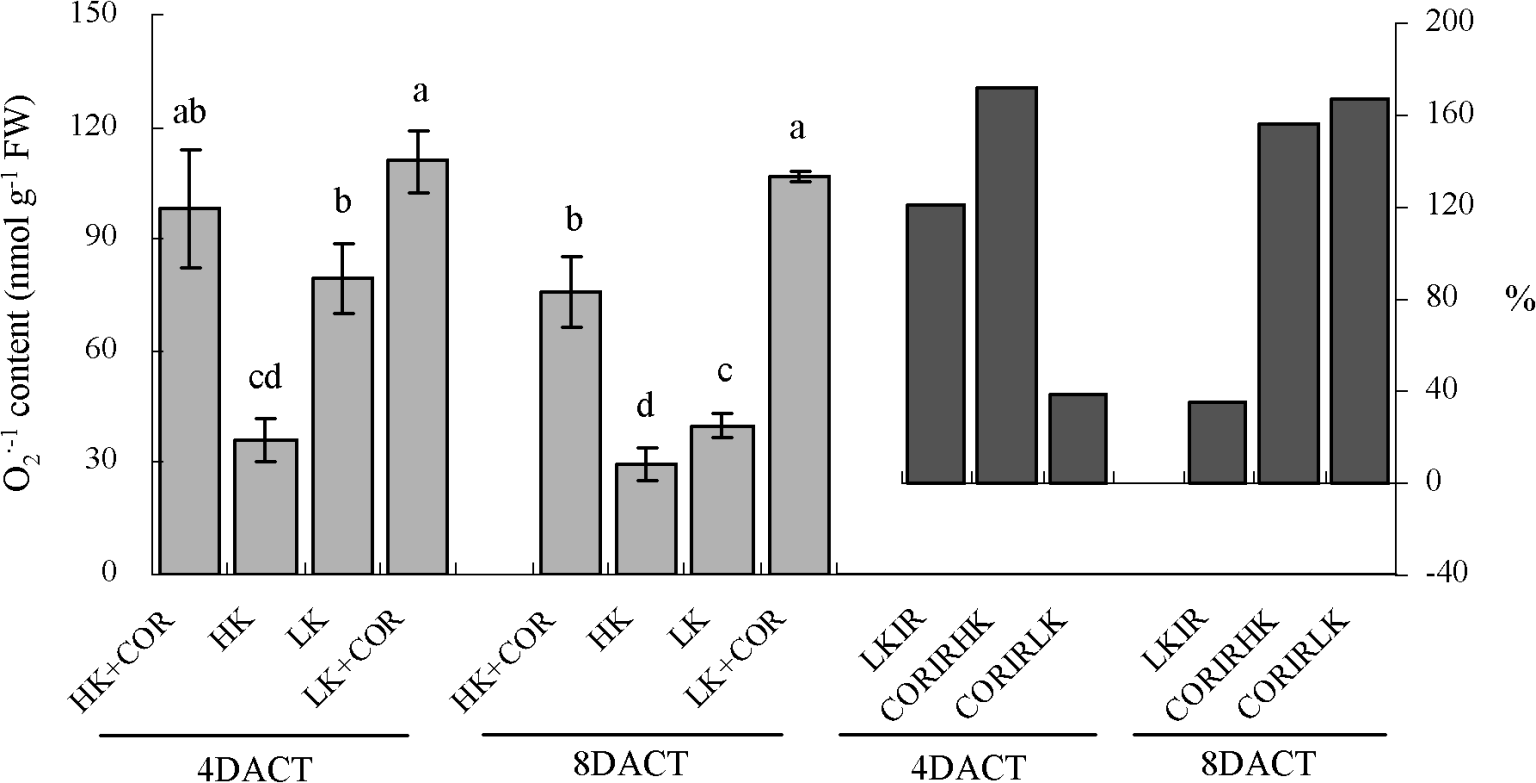
Effects of COR treatment on superoxide anion (O2^•-^) concentrations in roots under high K (2.5 mM) or low K (0.05 mM) conditions. Emerging seedlings were grown in solution for 3DAKT and subsequently transferred to fresh solutions without (0 nM) or with (10 nM) COR. O_**2**_^**·-1**^ concentrations in roots were determined at 4DACT (7DAKT) and 8DACT (11DAKT). Values are presented as means ± SD, n = 5. Bars with same letter are not significantly different at p≤0.05 as determined using Duncan’s multiple range test. Values showed by the right subfigure were, respectively, LK induced rangeability (LKIR) in comparison with HK, COR induced rangeability under HK(CORIRHK) and COR induced rangeability under LK (CORIRLK).

Prolonged K treatment from 7DAKT to 11DAKT resulted in a non-significant decrease in O_2_^·-^ levels under HK and a significant decrease in O_2_^·-^ levels under LK. LK increased O_2_^·-1^ levels by 1.2- and 0.3-fold, compared with HK. COR treatment increased O_2_^·-^ levels by 1.7- and 1.6-fold under HK and by 0.4- and 1.7-fold under LK at 4DACT and 8DACT, respectively. COR treatment increased relative O_2_^•-^ levels following prolonged K deficiency, compared with non-COR treatment (Fig 4).

**Fig 5:**
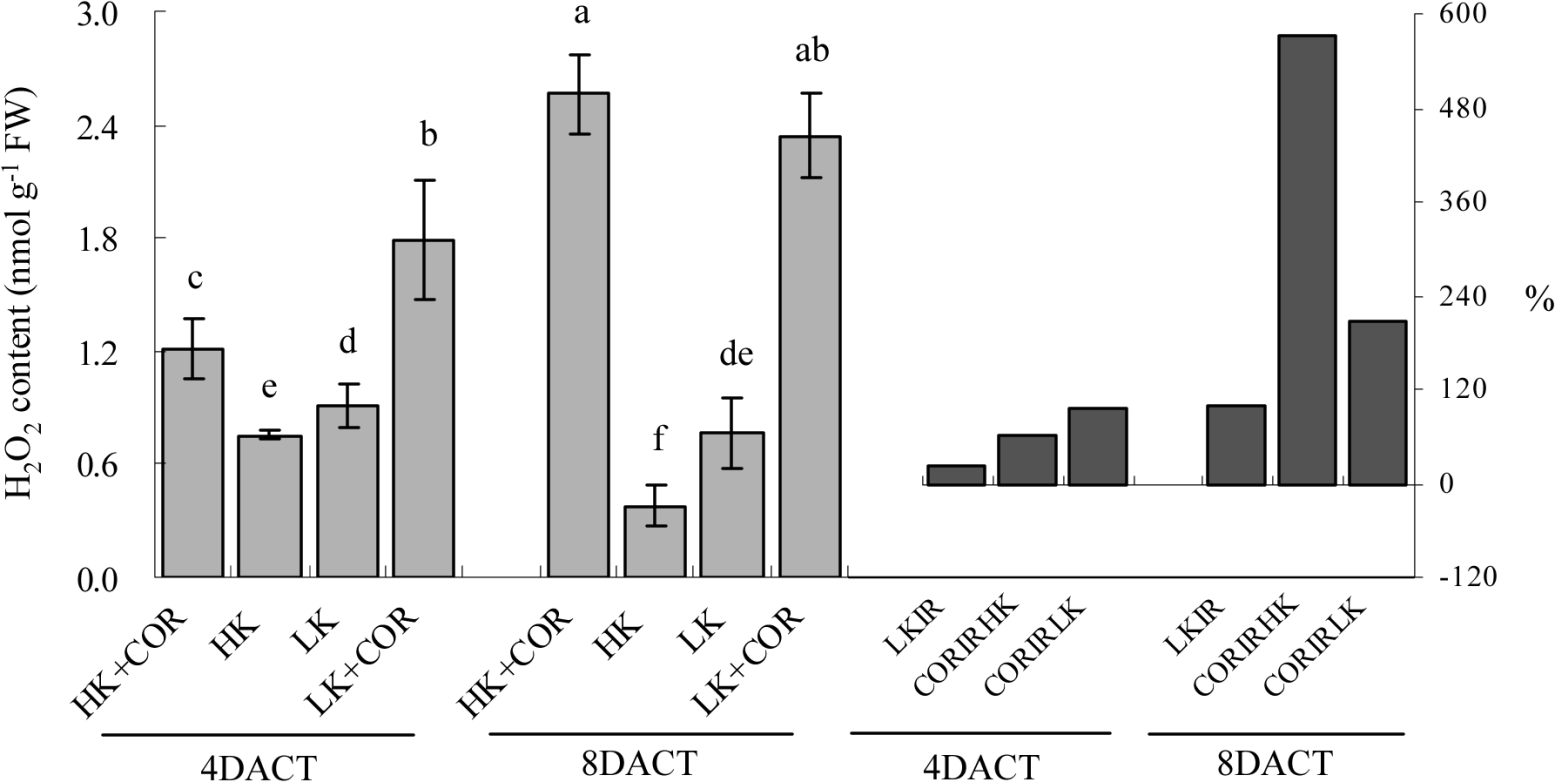
Effects of COR treatment on hydrogen peroxide (H_2_O_2_) concentrations in roots under high K (2.5 mM) or low K (0.05 mM) conditions. Emerging seedlings were grown in solution for 3DAKT and subsequently transferred to fresh solutions without (0 nM) or with (10 nM) COR. H_**2**_O_**2**_ concentrations in roots were determined at 4DACT (7DAKT) and 8DACT (11DAKT). Values are presented as means ± SD, n = 5. Bars with same letter are not significantly different at p≤0.05 as determined using Duncan’s multiple range test. Values showed by the right subfigure were, respectively, LK induced rangeability (LKIR) in comparison with HK, COR induced rangeability under HK(CORIRHK) and COR induced rangeability under LK (CORIRLK).

Prolonged K treatment from 7 to 11DAKT led to significantly decreased H_2_O_2_ levels under HK, and non-significant effects on the H_2_O_2_ levels were observed under LK. LK caused the H_2_O_2_ levels to increase by 0.2-% and 1-fold %, compared with HK. With prolonged COR treatment from 4 to 8DACT, the H_2_O_2_ levels increased by 0.6 and 5.7-fold under HK and by 1.0- and 2.1-fold under LK. Following treatment with both HK and COR, the H_2_O_2_ levels were markedly lower at 4DACT/7DAKT than at 8DACT/11DAKT (Fig 5).

**Fig 6:**
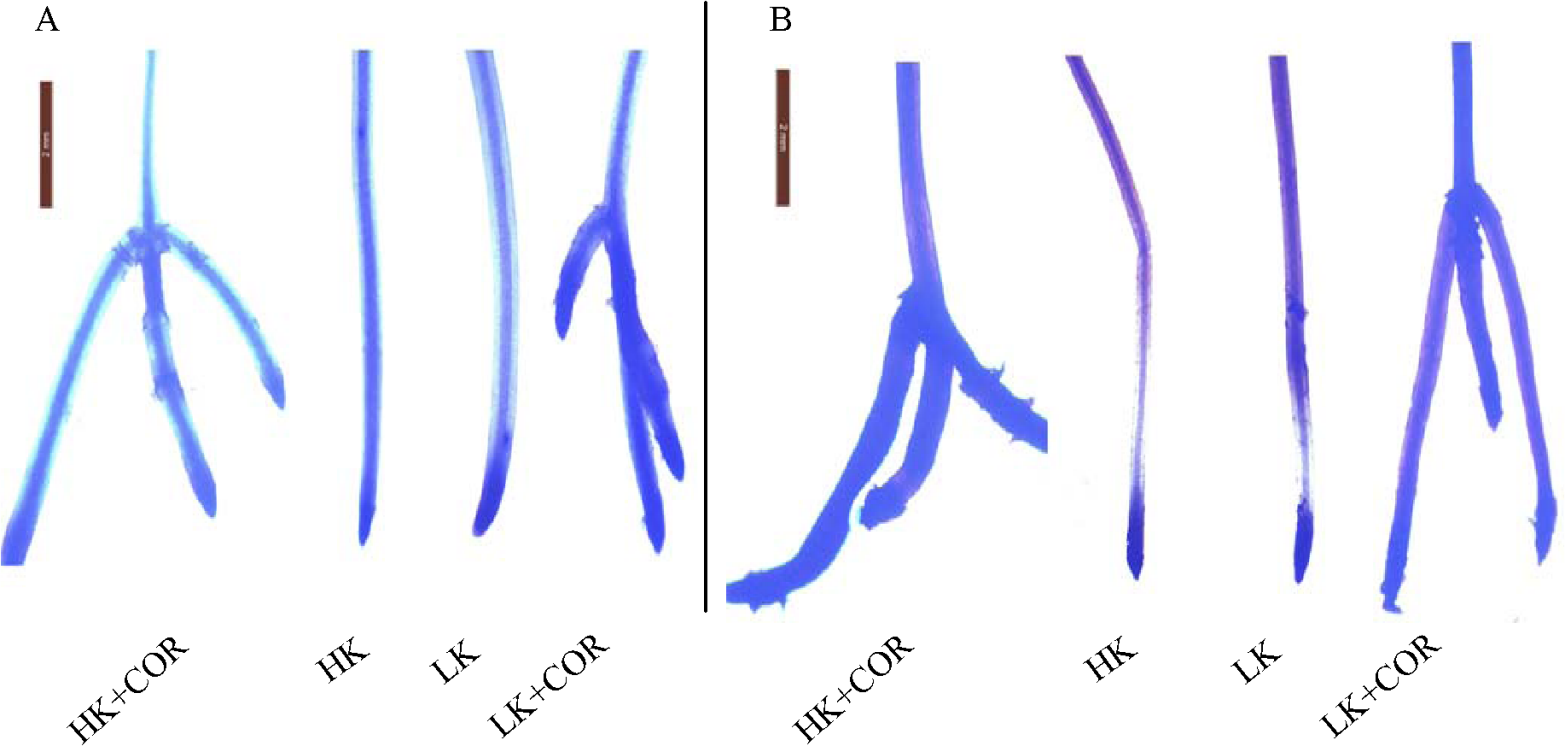
Effects of COR treatment on superoxide anion (O2^•-^) (A) and hydrogen peroxide (H_2_O_2_) (B) concentrations in root tips as shown by NBT and DAB staining, respectively, under high K (2.5 mM) or low K (0.05 mM) conditions. Emerging seedlings were grown in solution for 3DAKT and subsequently transferred to fresh solutions without (0 nM) or with (10 nM) COR. DAB and NBT staining of root tips were assessed at 8DACT (11DAKT). The photographs show representative plant roots from at least 8 plants analysed for each treatment.

COR at 10 nM shortened root elongation, but enhanced lateral root formation and made later lateral root closer to their parent root apex (Fig 6), which was similar with the previous study ^22^. NBT histochemical staining of O_2_^•-^ and DAB staining of H_2_O_2_ showed that LK increased ROS accumulation in the root tips compared with HK, and COR enhanced ROS accumulation under both HK and LK. These results are consistent with the trends observed in ROS concentrations (Fig 6).

The above results demonstrated that LK significantly promoted ROS production in comparison with HK, and COR significantly enhanced it independently of K levels.

### Effects of COR treatment on antioxidant enzyme activities under HK and LK

**Fig 7:**
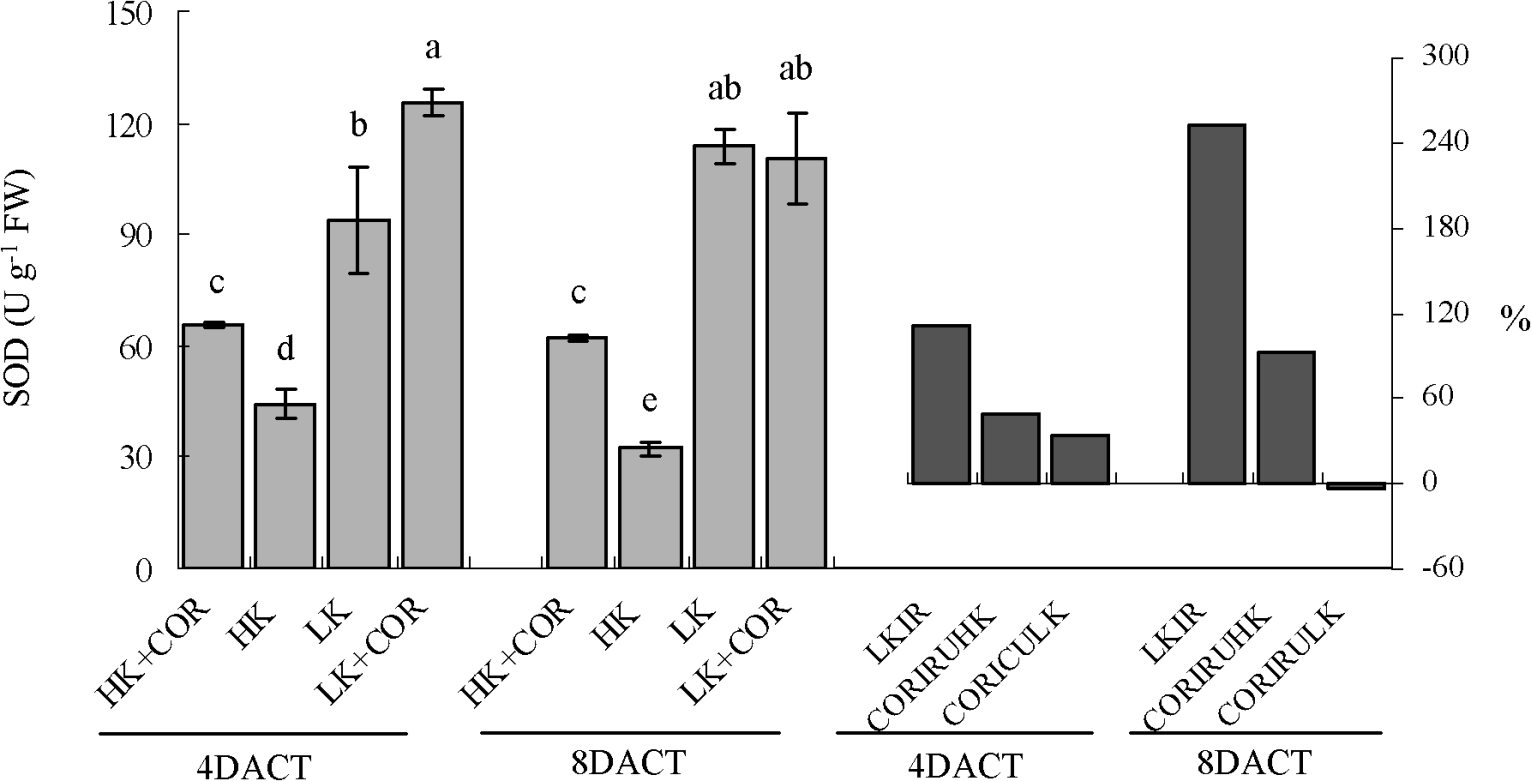
Effects of COR treatment on SOD activity under high K (2.5 mM) or low K (0.05 mM) conditions. Emerging seedlings were grown in solution for 3DAKT and subsequently transferred to fresh solutions without (0 nM) or with (10 nM) COR. SOD activity levels were determined at 4DACT (7DAKT) and 8DACT (11DAKT). Values are presented as means ± SD, n = 5. Bars showing same letter are not significantly different at p≤0.05 as determined using Duncan’s multiple range test. Values showed by the right subfigure were, respectively, LK induced rangeability (LKIR) in comparison with HK, COR induced rangeability under HK(CORIRHK) and COR induced rangeability under LK (CORIRLK).

**Fig 8:**
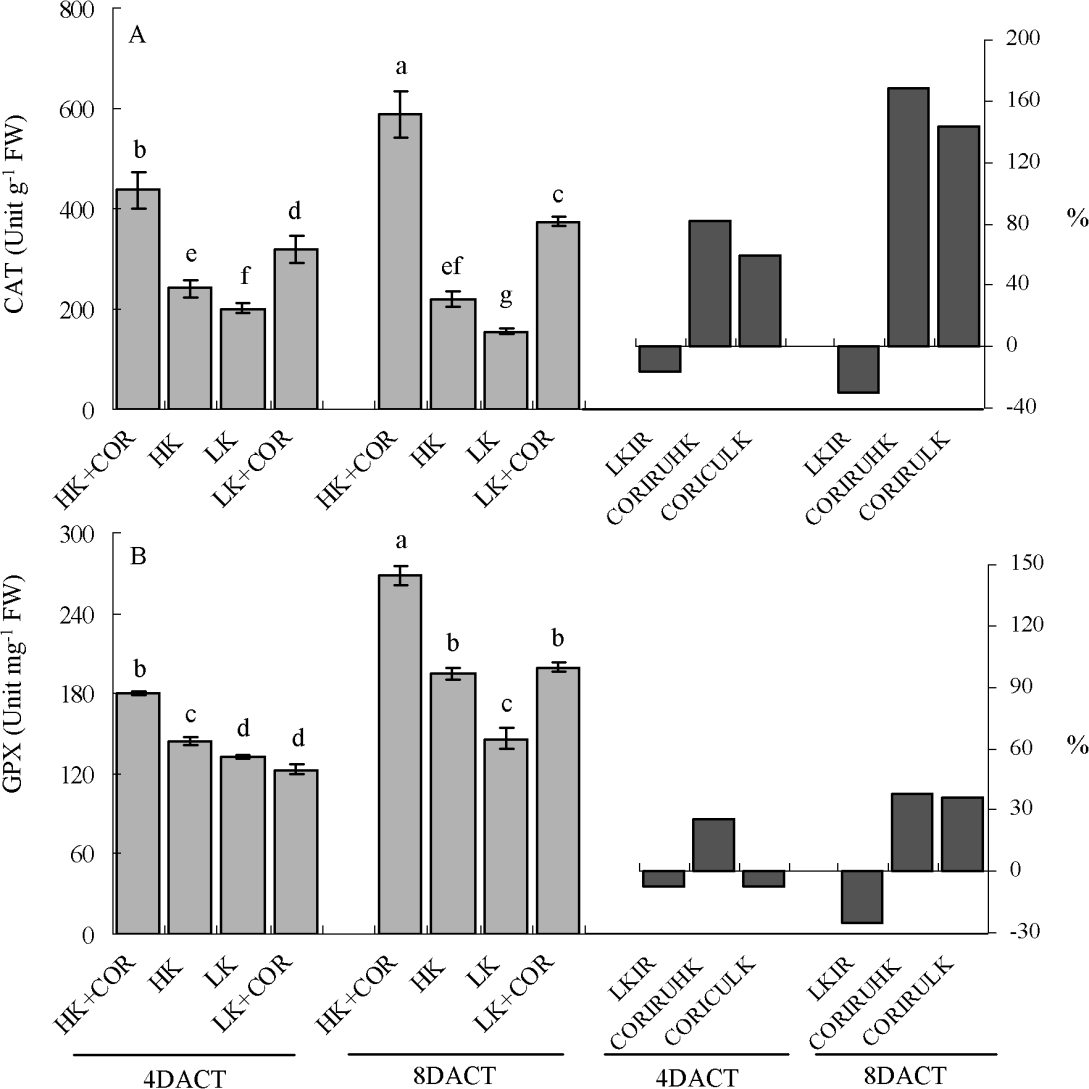
Effects of COR treatment on activities of antioxidant enzymes (CAT and GPX) associated with hydrogen peroxide (H_2_O_2_) decomposition under high K (2.5 mM) or low K (0.05 mM) conditions. Emerging seedlings were grown in solution for 3DAKT and subsequently transferred to fresh solutions without (0 nM) or with (10 nM) COR. Antioxidant enzyme activities were determined at 4DACT (7DAKT) and 8DACT (11DAKT). Values are presented as means ± SD, n = 5. Bars with same letter are not significantly different at p≤0.05 as determined using Duncan’s multiple range test. Values showed by the right subfigure were, respectively, LK induced rangeability (LKIR) in comparison with HK, COR induced rangeability under HK(CORIRHK) and COR induced rangeability under LK (CORIRLK).

**Fig 9:**
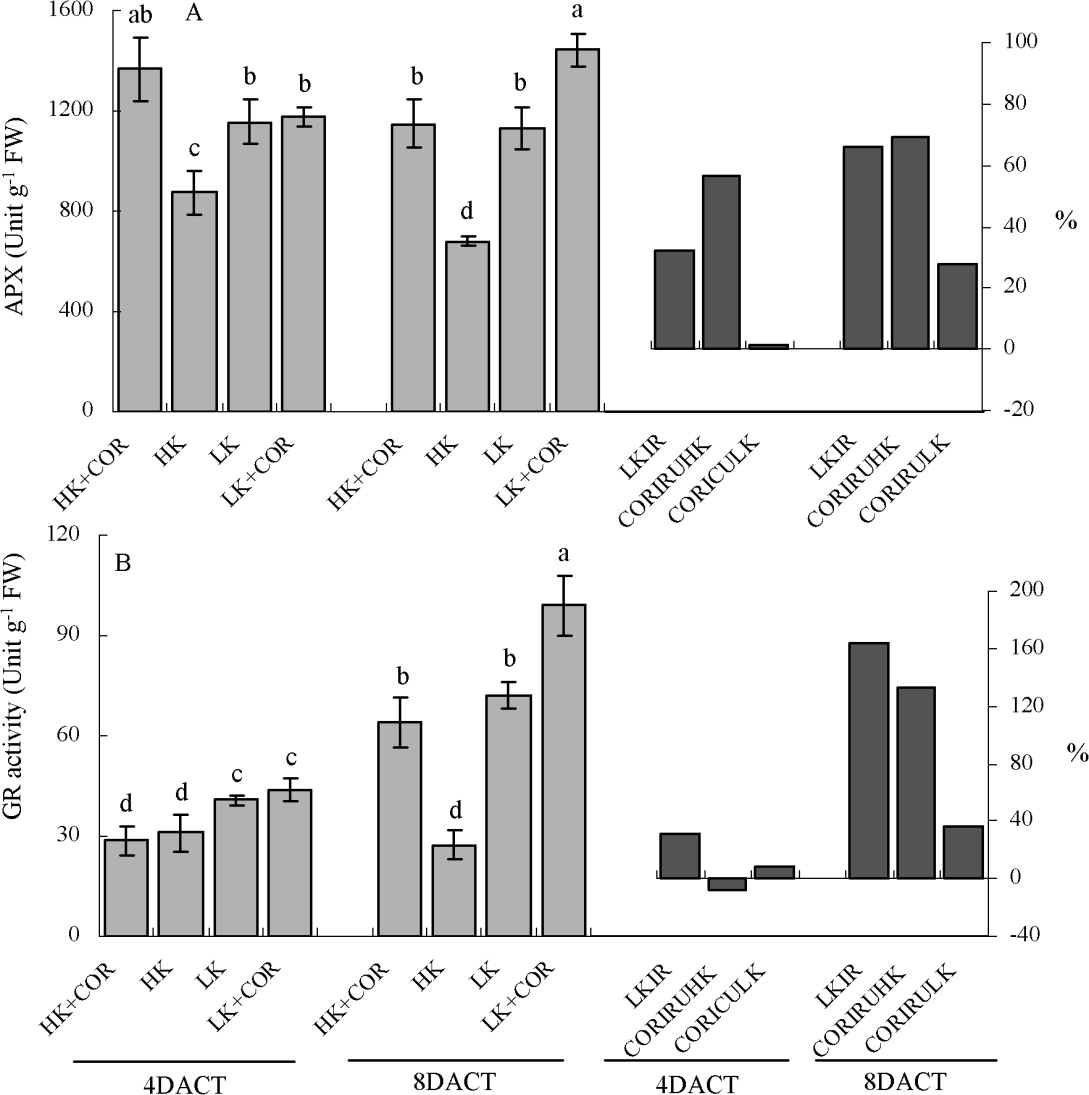
Effects of COR treatment on activities of antioxidant enzymes (APX and GR) associated with hydrogen peroxide (H_2_O_2_) decomposition under high K (2.5 mM) or low K (0.05 mM) conditions. Emerging seedlings were grown in solution for 3DAKT and subsequently transferred to fresh solutions without (0 nM) or with (10 nM) COR. Antioxidant enzyme activities were determined at 4DACT (7DAKT) and 8DACT (11DAKT). Values are presented as means ± SD, n = 5. Bars with same letter are not significantly different at p≤0.05 as determined using Duncan’s multiple range test. Values showed by the right subfigure were, respectively, LK induced rangeability (LKIR) in comparison with HK, COR induced rangeability under HK(CORIRHK) and COR induced rangeability under LK (CORIRLK).

Prolonged K treatment from 7 to 11DAKT led to the increased activity of the O_2_^•-^decomposing enzyme SOD from 1.1- to 2.5-fold under LK compared with HK. With respect to the H_2_O_2_-decomposing enzymes, CAT activity decreased from 16% to 30% and GPX activity decreased from 7% to 25%. Conversely, APX activity increased from 32% to 66% and GR activity increased from0.3- to 1.6-fold. Under HK, SOD, CAT, APX and GR showed higher levels of activity at 7DAKT than at 11DAKT (Figs 7–9).

At 4DACT/7DAKT, COR significantly increased SOD activity by 49% and 34% and CAT activity by 82% and 59% under HK and LK, respectively. GPX activity increased by 25% and APX activity did by 54% under HK. There were no significant differences in GR activity between the COR and non-COR treatments under HK and among the GPX, APX and GR activity levels under LK (Figs 8 and 9).

At 8DACT/11DAKT, COR significantly increased SOD activity by 99% under HK, and CAT activity increased by 1.7- and 1.4-fold, GPX activity increased by 38% and 36%, APX activity increased by 69% and 28%, and GR activity increased by 1.3- and 0.4-fold under HK and LK, respectively. There was no significant difference in SOD activity between the COR and non-COR treatments under LK (Figs 7–9).

All these results indicated that LK significantly affected the activities of antioxidant enzymes in comparison with HK, and that COR influenced them with K- and treating time-dependent manner.

### Effects of COR treatment on ROS scavenging by non-enzymatic antioxidants under HK and LK

**Fig 10:**
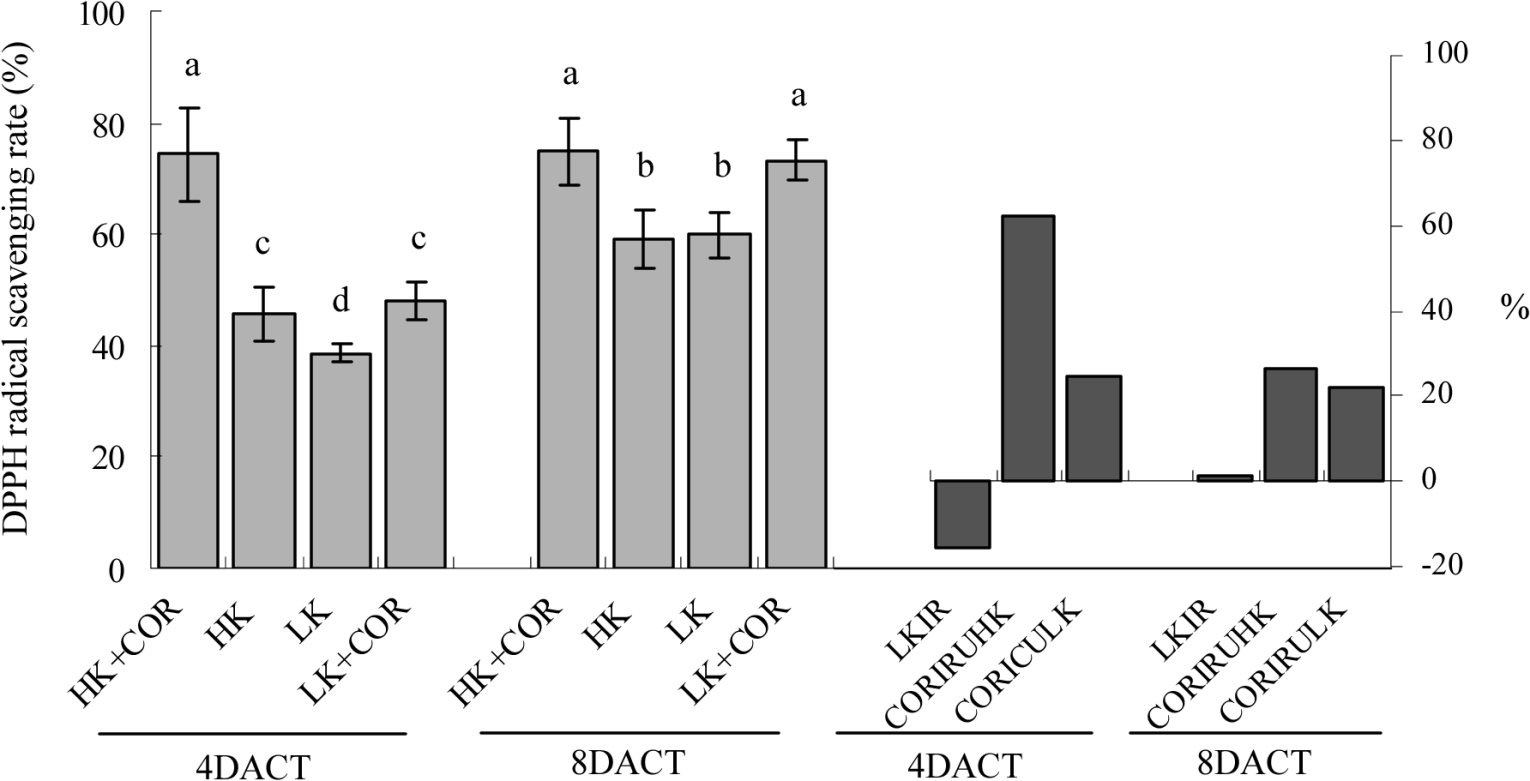
Effects of COR treatment on DPPH radical-scavenging rate under high K (2.5 mM) or low K (0.05 mM) conditions. Emerging seedlings were grown in solution for 3DAKT and subsequently transferred to fresh solutions without (0 nM) or with (10 nM) COR. The DPPH radical-scavenging rates were determined at 4DACT (7DAKT) and 8DACT (11DAKT). Values are presented as means ± SD, n = 5. Bars with same letter are not significantly different at p≤0.05 as determined using Duncan’s multiple range test. Values showed by the right subfigure were, respectively, LK induced rangeability (LKIR) in comparison with HK, COR induced rangeability under HK(CORIRHK) and COR induced rangeability under LK (CORIRLK).

ROS scavenging by non-antioxidant enzymes was expressed as DPPH-radical scavenging activity. At 7DAKT, LK significantly decreased DPPH radical-scavenging activity by 16% compared with HK; however, its activity was unaffected by LK at 11DAKT. COR treatment increased DPPH radical-scavenging activities by 62% and 27% under HK and by 25% and 22% under LK at 4DACT and 8DACT, respectively (Fig 10). This result illustrated that only short term LK significantly reduced DPPH radical-scavenging activity, and that COR significantly increased it with K-independent manner.

## Discussion

ROS production is activated under abiotic stresses [19], including cold temperature [20], ozone [21], wounding [22], drought [23], salt stresses [24], and nutrient starvation such as Zn [25], Mg [26], phosphorus and nitrogen [10,27]. ROS accumulation accelerated membrane lipid peroxidation [2,28], which is generally considered to be a biomarker of an extensive oxidative stress [29,30]. However, there is not necessarily a positive correlation between H_2_O_2_ and lipid peroxidation. For example, H_2_O_2_ concentrations for inducing programmed cell death could be ranged for 20-fold in mammalian species [31]; under boron toxicity, both parameters have been shown to increase significantly in the tomato [32], apple rootstock [33] and grape [34], but the synergistic effects have not been observed in barley [35]; lipid peroxidation was decreased in Mg-deficient plants with significant increase in H_2_O_2_ [26]. To increase resistance to oxidative damage and to improve plant growth, plants require high levels of antioxidants [36]. The enhanced activity of antioxidant enzymes and antioxidant ability of non-enzymatic substances has typically been associated with the acclimation to elevated ROS levels and increased stress tolerance [37].

### K deficiency and COR treatment lead to ROS homeostasis and oxidative stress

Probably depending on plant species and treatment time, effects of potassium deficiency on ROS contents varied. Potassium deficiency increased ROS in *Arabidopsis* leaves and roots [9,10,27], tomato roots [11], soybean and maize leaves [24,38] and cotton roots (Figs 4–6). However, Hafsi *et al*. observed that potassium deficiency significantly decreased ROS level in barley leaves [8], and Tewari *et al*. observed no significant effect of potassium deficiency on H_2_O_2_ content in, respectively, maize and mulberry leaves [39].

K deficiency enhances O_2_^•-^ production and it was stimulated by abiotic stresses, such as cold, drought and Zn deficiency [7]. It might account for that there was significantly higher levels of O_2_^•-^ production at 7DAKT than at 11DAKT under LK (Fig 4), because the seedling roots required adaption to their new environments, which is most likely involved stress, e.g., osmotic stress, when emerging seedlings were initially transferred to culture solutions. The stress caused by this environmental adaptation also led to reduced TTC reduction levels and increased MDA levels under HK at 7DAKT in comparison with 11DAKT (Figs 1 and 2).

Although potassium derivation induced different ROS homeostasis, it led to accumulation of MDA [8,11,24,39], suggesting that K deficiency resulted in oxidative damages to lipids and constitute oxidative stress. In our work, in addition to increased MDA content and initially high electrolytic leakage (Fig 2), K deficiency further decreased TTC reduction in cotton roots (Fig 1); this is a useful qualitative indicator of cell viability [40] and is a good estimator of root metabolic activity [41–43]. Therefore, K deficiency not only induced ROS accumulation but also resulted in oxidative stress reflected by significantly enhanced MDA content and lowered TTC reduction in cotton roots.

COR is structurally and functionally similar to jasmonate [13]. Jasmonate exposure has been shown to increase endogenous ROS production in *Ricinus communis* and *Artemisia annua* leaves [29], but simultaneously inhibits lipid peroxidation under normal and boron stress conditions [29]. COR may play similar function in cotton in which it induced ROS through the plant defence mechanism. In this study, we observed that COR increased O_2_^•-^ and H_2_O_2_ production under either HK or LK, and H_2_O_2_ production was gradually increased with prolonged treatment times in the present study, but ameliorated oxidative stress reflected by significantly lowered MDA content (only at 8DACT) and enhanced root vitality (Figs 1 and 2). COR-induced ROS increase in cotton roots may originate from mitochondria and ROS-generating heme proteins, not excluding from other enzymes such as oxalate oxidase and amine oxidase in the apoplast. A previous study showed that COR regulated PS II and ROS-generating heme proteins and further enhanced ROS in tomato [44]. A significantly greater reduction in TTC levels was induced by COR under HK compared with LK (Fig 1), suggesting that the effects of COR were dependent on the K levels. The origin of ROS during COR exposure appears to be complex and highly regulated by requisite time-dependent coordinated up-and down-regulations of differently located enzymes which produce or scavenge ROS. This progress may be controlled at both transcriptional and post-transcriptional levels [45].

### Responses of non-enzymatic and enzymatic antioxidant capacities to K deficiency or COR treatment

The response of the antioxidant system has been studied under potassium deficiency in different species and under different starvation periods ranging from short time (1–7 d) to long time (14–60 d) [8,11,24,38]. The accumulation of oxidative damage in potassium-starved cotton seedling roots indicated an imbalance between ROS production and the antioxidant systems. To study non-enzymatic role in ROS scavenging, we analysed DPPH-radical scavenging activities and found them significantly decreased initially (Fig 10). For enzymatic role, we analysed the enzyme activity of SOD, APX, GR, CAT and GPX. K deficiency increased antioxidant activities of SOD, APX and GR in roots, but it has been demonstrated to inhibit the activities of CAT and GPX (Figs 7–9). The increased SOD activity was insufficient to scavenge the excess O_2_^•-^ production (Fig 5). The increase of APX and GR could be compensated for the lower CAT and GPX activity, avoiding a toxic accumulation of H_2_O_2_ in the cells. Notably, the changing pattern of antioxidant enzymatic activity and non-enzymatic ability varied among different studies in plant response to potassium deficiency [8,11,38].

COR has been shown to increase the resistance of rice to drought by enhancing the activities of CAT, SOD, APX and GR and inducing the accumulation of polypeptides [14]. COR treatment also alleviated salt stress in cotton by enhancing the activities of CAT, SOD, GPX and GR and the DPPH-radical scavenging capacity [16]. The findings from these two previous studies suggest that an increased antioxidant capacity leads to the alleviation of ROS production and the promotion of plant growth; however, these studies did not report the detailed ROS production [14,16]. Subsequently, Xie *et al*. showed low concentration of COR (10 nM) decreased H_2_O_2_ content in cotton roots and increased seedling weight under salt stress [46]. In this present study, COR alleviated potassium deficiency showed by increasing root viability and decreasing root lipid peroxidation with prolonged treatment time concomitant with high levels of ROS production and the increased activities of most antioxidant enzymes, acting in similar manner with jasmonate in *Artemisia annua* L plants under boron toxicity [29].

In detail, DPPH-radical scavenging activities was increased by COR under HK and LK, similarly with jasmonate’s role [47], respecting non-enzymatic antioxidant capacity. Considering antioxidant enzymes, Jung *et al*. reported that jasmonate increased total activity of SOD, CAT, GPX and GR after 7 days of treatment in *A*. *thaliana* leaves [47]. Soares *et al*. reported transiently increased activity of CAT and GPX, decreased SOD activity [48] and increased APX after *Ricinus communis* leaves’ treatment with jasmonate [47]. COR induced an obviously higher increase in APX activity concomitant with the substantial activation of GR in the roots at 8DACT under HK than those observed under LK (Fig 9A and 9B). COR also significantly and gradually increased CAT and GPX activity levels in the cotton roots (Fig 8A and 8B). Interestingly, total SOD activity was significantly increased at 4DACT and not significantly affected at 8DACT by COR under LK (Fig 7). The activity increase in H_2_O_2_-decomposing enzymes like APX, GPX and CAT and antioxidant non-enzymatic substances does not inhibit the further H_2_O_2_ content increase in cotton roots.

In conclusion, the results of this present study show that low K concentrations in the culture medium lead to the accumulation of oxidative damage in the roots of cotton seedlings, although induce the responses of antioxidant enzymes (SOD, APX and GR), simultaneously compose oxidative stress. In addition, COR treatment induces general antioxidant system (non-enzymatic and most enzymatic capability) responses and significantly increases the production of ROS by a large margin in the treated cotton seedlings, which does not induce but alleviate oxidative stress.

## Materials and Methods

### Plant materials and culture conditions

The cotton cultivar DP 99B, being sensitive to K deficiency [17], was used in this study. The experiments were conducted in a growth chamber under the following conditions: 30/25°C, 14/10 h light/dark period, and 450 μmol m^-2^ s^-1^ light. The seeds were surface sterilised with 10% H_2_O_2_ for thirty min, washed with tap water three times, and soaked for 12 h in tap water. The soaked seeds were germinated and emerged in wet sand. Only those seedlings that emerged were transferred to a culture solution containing 2.5 mM KCl or 0.05 mM KCl, and this time point was denoted as 0 day after K treatment (DAKT). Sodium ions were provided using NaCl to seedlings exposed to the low-K conditions. In addition to different concentrations of KCl, other mineral nutrients were also added to the culture solution, including 2.5 mM Ca(NO_3_)_2_, 1 mM MgSO_4_, 0.5 mM NH_4_H_2_PO_4_, 2 mM NaCl, 2×10^–4^ mM CuSO_4_, 1×10^–3^ mM ZnSO_4_, 0.1 mM EDTA-FeNa, 2×10^–2^ mM H_3_BO_3_, 5×10^–6^ mM (NH_4_)_6_Mo_7_O_24_, and 1×10^–3^ mM MnSO_4_. After 3 days (i.e., 3DAKT), COR was added at a final concentration of 10 nM (this concentration was suitable as described in the previous study ^22^), and this time point was denoted as 0 day after COR treatment (DACT).

The culturing pot was 20×13×15 cm in size and contained 4 L of solution. The solution was continuously aerated and adjusted daily to pH 7.0. At 4DACT/7DAKT and 8DACT/11DAKT, the roots of the uniform seedlings under the different treatments, except for the coarse taproot (approximately 4–5 cm from the junction of the stem and taproot), were washed three times with deionised water to remove surface-adhered electrolytes and were subsequently sampled to determine the following physiological and biochemical properties.

### Root vitality

Root vitality was examined as the root triphenyl tetrazolium chloride (TTC) reduction in tissues following treatment with red-coloured insoluble triphenylformazan [49]. TTC reduction was determined using the modified method of Lutts *et al*. Root segments of approximately 1 cm [500 mg fresh weight (FW)] were incubated for 24 h in the dark at 30°C with 5 mL of 0.6% (w/v) TTC solution dissolved in 100 mM phosphate buffer (pH 7.4). The root segments were subsequently recovered on filter paper, washed with deionised water and blotted onto blotting paper. Water-insoluble red formazan was extracted from the tissues at 85°C for 20 min in 5 mL of 95% (v/v) ethanol. The absorbances of the extracts were measured at 485 nm, and these values were used to calculate the root vitalities (i.e., absorbances at 485 nm g^-1^ FW).

### Lipid peroxidation

Lipid peroxidation was determined according to measurements of malondialdehyde (MDA) concentrations by the thiobarbituric acid (TBA) reaction. The amount of MDA-equivalent TBA-reactive substance (TBARS) was derived from the difference in absorbance at 532 and 600 nm using an extinction coefficient of 155 mM cm^-1^.

### Relative electrolyte leakage

Relative electrolyte leakage was assessed according to Lutts *et al*. (1996). The root segments were placed in closed vials containing 10 mL of deionised water and incubated at 35°C on a rotary shaker for 2 h. Subsequently, the electrical conductivity of the solution (Lt) was determined. The samples were autoclaved at 120°C for 20 min, and the final electrical conductivity (L_0_) was obtained after equilibration at 25°C. The relative electrolyte leakage was defined as (L_t_/L_0_)×100.

### ROS determination

ROSs were estimated by the total O_2_^•-^ and H_2_O_2_ concentrations in the roots and their histochemical staining intensities.

Superoxide anion (O_2_^•-^) levels were determined according to Elstne *et al*. (1976) [50]. The root samples were homogenised in 50 mM phosphate buffer and centrifuged at 10000×*g* at 4°C for 15 min, and the supernatants were collected as O_2_^•-^ extracts. A mixture of 0.5 mL extract, 0.5 mL 50 mM phosphate buffer (pH 7.8), and 0.1 mL 10 mM hydroxylamine hydrochloride was incubated at 25°C for 1 h, and subsequently, 1 mL 58 mM *p*-aminobenzene sulphonic acid and 1 mL 7 mM α-naphthylamine were added. The final mixture was incubated at 25°C for 20 min, and absorbance was measured at 530 nm. The standards were prepared using NaNO_2._

Hydrogen peroxide (H_2_O_2_) levels were determined using a modified ferrous ammonium sulphate/xylenol orange (FOX) method [51]. Frozen roots were homogenised in cold, pure acetone and centrifuged at 10000×*g* at 4°C for 15 min, and the supernatants were collected as H_2_O_2_ extracts. The assay mixture (after the addition of the sample) contained 200 μM ferrous ammonium sulphate, 100 μM sorbitol, and 100 μM xylenol orange in 25 mM H_2_SO_4_. After incubation at 30°C for 30 min, absorbance was measured at 560 nm. The standards were prepared using a 30% dilution of reagent-grade H_2_O_2_. The H_2_O_2_ concentration of this reagent was calibrated using its absorbance at 240 nm and an extinction coefficient of 43.6 M^-1^ cm^-1^.

Two staining methods were applied to study ROS accumulation in the root tips according to a previous report [52]. Nitroblue tetrazolium (NBT) staining was used for the specific detection of O_2_^•-^ in the roots according to Mellersh *et al*. (2002) [53]. Diaminobenzidine (DAB) staining was used for the histochemical detection of H_2_O_2_. The sites of O_2_^•-^ and H_2_O_2_ accumulation stained dark blue and brown, respectively.

### Activities of antioxidant enzymes

The root samples were ground in liquid nitrogen using a mortar and pestle, extracted in 5 mL of 100 mM sodium phosphate buffer (pH 7.5) containing 1 mM EDTA, and centrifuged at 15000×g for 20 min at 4°C. The supernatant was used as an enzyme source [54].

SOD (EC 1.15.1.1) activity was measured using the NBT photochemical method. One unit of SOD activity was defined as the amount of enzyme required for the 50% inhibition of the rate of NBT reduction at 560 nm, and SOD activity was expressed as unit g^-1^ FW. CAT (EC 1.11.1.6) activity was determined as the decrease in absorbance at 240 nm for 3 min following the decomposition of H_2_O_2_. The 3-mL reaction mixture contained 100 mM sodium phosphate (pH 7.0), 0.5 mL enzyme extract and 10 mM H_2_O_2_. GPX (EC 1.11.1.7) activity was determined using guaiacol at 470 nm according to Polle *et al*. [55]. The 3-mL reaction mixture contained 100 mM potassium phosphate (pH 6.5), 16 mM guaiacol, 10 mM H_2_O_2_ and 0.05 mL enzyme extract. The reaction was initiated upon the addition of the enzyme extract.

APX (EC 1.11.1.11) activity was measured according to Asada (1984) [6] as the decrease in absorbance at 290 nm during the oxidation of ascorbate by H_2_O_2_. The 3-mL reaction mixture contained 50 mM sodium phosphate (pH 7.0), 0.5 mM ascorbate, 0.25 mM H_2_O_2_ and 0.5 mL enzyme extract. GR (EC 1.6.4.2) activity was determined as the oxidation of NADPH at 340 nm according to Rao *et al*. [56]. The 3-mL reaction mixture contained 100 mM sodium phosphate (pH 7.8), 1 mM EDTA, 0.2 mM NADPH, 0.5 mM GSSG and 0.5 mL enzyme extract. The assays were initiated upon the addition of NADPH.

The activities of all antioxidant enzymes were represented by the FWs, and one unit of CAT, GPX, APX or GR activity was defined as 0.01 unit of the OD value decrease per min, expressed as unit g^-1^ FW for CAT, APX and GR or unit mg^-1^ FW for GPX.

#### DPPH (1, 1-diphenyl-2-picrylhydrazyl) radical-scavenging activity

The root samples (0.3 g FW) were homogenised at 4°C in 4.0 mL of absolute ethanol using a mortar and pestle and centrifuged at 10000×*g* for 20 min at room temperature. A 1-mL aliquot of the supernatant was mixed with 1 mM DPPH ethanol solution (1 mL) and 100 mM acetate buffer (pH 5.5; 3 mL). After standing for 15 min, the absorbance of the mixture was measured at 517 nm [57].

### Statistical analysis

Each experiment was repeated three times with similar results. Each pot was treated as one replicate, and all treatments were replicated five times. Five different root samples of each treat at each time were used for determination of above each physiological index. The data were statistically analysed using the analysis of variance [37] according to a simple randomised block design. The mean values were statistically compared using Duncan’s multiple range test at p≤0.05 significance level. The photographs show the representative plant roots selected from at least 8 plants analysed for each treatment.
